# Ferrous sulfate oral solution in young children with iron deficiency anemia: An open‐label trial of efficacy, safety, and acceptability

**DOI:** 10.1111/ped.14237

**Published:** 2020-07-09

**Authors:** Lidia Pachuta Węgier, Maciej Kubiak, Agata Liebert, Thierry Clavel, Agnès Montagne, Aline Stennevin, Sandrine Roye, Asmaa Boudribila

**Affiliations:** ^1^ Lidia Pachuta Węgier Medical Services Lublin Poland; ^2^ Tolek Clinic for Children Lesznowola Poland; ^3^ TCC Consulting Revel France; ^4^ Clinical Development Department Institut de Recherche Pierre Fabre CRDPF Toulouse France

**Keywords:** ferrous sulfate, infant, iron deficiency anemia, preschool child

## Abstract

**Background:**

This study evaluated the efficacy, safety, and acceptability of a new ferrous sulfate oral solution (Tardyferon® 20 mg/mL) in young children with mild or moderate iron deficiency anemia (IDA).

**Methods:**

This was a multicenter, national, single‐arm, open‐label study. Children aged 6–53 months presenting with mild or moderate IDA (i.e., blood hemoglobin (Hb) ranging from 7.0 to 10.9 g/dL and serum ferritin <12 ng/mL) were eligible for inclusion. The ferrous sulfate heptahydrate solution (2 mg/kg/day) was administered orally for 3 months. If normalization of either Hb or ferritin was not achieved at month 3 the treatment was continued for another 3 months.

**Results:**

Of the 100 children screened, 21 aged 6–17 months were included and received the study treatment, and 19 were analyzed for hematologic outcomes at month 3. Only one patient continued treatment for the additional 3 months. At month 3, mean ± SD Hb and ferritin levels were 12.0 ± 0.7 g/dL and 31.5 ± 19.4 ng/mL, respectively. Hemoglobin and ferritin levels were normalized in 95% (18/19) and 84% (16/19) of the patients, respectively. Treatment compliance and levels of satisfaction of both the parents and the investigators were high. Overall, 33.3% of patients (7/21) experienced at least one adverse event. Only one patient (4.8%) experienced a drug‐related adverse event (upper abdominal pain).

**Conclusions:**

A 2 mg/kg daily dose of the new oral ferrous sulfate heptahydrate solution provides substantial therapeutic benefit with high levels of tolerability in young children who have mild or moderate IDA.

Iron deficiency (ID) and iron‐deficiency anemia (IDA) are a worldwide concern. With 750 million children affected around the world, IDA is the most common nutritional disorder occurring during childhood.^1^ Among children in the developing world, ID is the most common single‐nutrient deficiency caused by insufficient intake. Despite a decline in prevalence, IDA remains a common cause of anemia in young children from industrialized countries. In 2011, the World Health Organization (WHO) estimates for children aged 6–59 months indicated a global prevalence of anemia of approximately 43%, ranging from around 22% in developed regions to around 62% in developing regions. Around 42% of cases of anemia in children can be attributed to ID.^2^

Iron deficiency and IDA impair the cognitive development and physical growth of infants and children, depress immune function, and increase morbidity from infectious diseases.^3^ Usually, ID evolves slowly and is not clinically apparent until the anemia is severe.^3^ Mild or moderate IDA may go unnoticed and is difficult to diagnose because IDA symptoms are infrequent and nonspecific, including pallor, irritability, poor appetite, fatigue, and lethargy.^4^, ^5^ Diagnosis is even more problematic in infants, for whom it is difficult to obtain blood samples in sufficient quantities for hematologic diagnosis.^5^

Iron supplementation to improve iron storage is the preferred treatment for IDA.^3^ Ferrous salts are preferred over ferric salts because of their absorption, which is about three times better.^6^ They are registered in the WHO model list of essential medicines as the most efficacious, safe, and cost‐effective iron supplements for the treatment of anemia in children^7^ and adults.^8^ The ferrous sulfate form, which is the most commonly prescribed, constitutes the treatment of choice.^6^ The recommended treatment for children is a liquid solution allowing the administration of a daily dose of 2 mg of elemental iron/kg.^3^ Pierre Fabre Médicament (France) has developed a new liquid formulation of ferrous sulfate heptahydrate (Tardyferon®; 20 mg/mL solution) to facilitate iron administration in individuals with difficulties swallowing tablets, e.g., children and elderly subjects. Moreover, as a graduated pipette is provided with the new ferrous sulfate solution, the dosage of the formulation can be adapted easily to the age and bodyweight of children. The present study aimed to assess the efficacy, tolerability, and acceptability of this ferrous sulfate solution in infants and young children suffering from mild or moderate IDA.

## Methods

### Study design

This multicenter, national, phase‐3, single‐arm, open‐label study was conducted in 10 health centers in Poland from June 2016 to January 2019. The study was conducted in accordance with the International Conference on Harmonization (ICH) Good Clinical Practice guidelines (CPMP/ICH/135/95) and the Declaration of Helsinki as amended. The study protocol was approved by the ethical committee for Lublin Medical Chamber and by the competent authority, according to national regulations. The parents / guardians of all patients gave their written informed consent at enrolment after having been informed of the methods, risks, and potential benefits of the trial.

### Participants

Infants, toddlers, and young children with mild or moderate IDA, defined by blood hemoglobin (Hb) levels ranging from 7.0 to 10.9 g/dL^9^ and serum ferritin concentrations <12 ng/mL,^10^ were eligible. As blood Hb and serum ferritin cut‐off levels to diagnose anemia are well established for young children aged between 6 months and 59 months,^9^, ^10^ and given that this study was to be conducted over a maximum period of 6 months, only children aged 6 months to 53 months (inclusive) could be included. Their bodyweight had to range between 7 and 20 kg. Children were excluded if they presented with anemia related to causes other than ID – inflammatory anemia, anemia due to marrow failure, hemoglobinopathies (sickle‐cell disease, thalassemia), hemolytic anemia, anemia due to acute hemorrhage, anemia related to chronic renal failure –, hemochromatosis or iron overload of secondary origin (blood transfusion), gastroduodenal ulcer, inflammatory bowel disease, any digestive disease that could modify iron absorption, or pica. Children were also excluded if they had received oral or parenteral iron treatment in the 3 weeks prior to the screening visit, if they had a history of hypersensitivity to at least one of the components of the tested treatment or a history of intolerance to oral iron derivatives, or if they needed a long‐term treatment known to modify iron absorption.

### Treatment

Given the consequences of IDA, it would not have been ethical to maintain young children suffering from IDA on a placebo. Consequently, each included patient was treated on an outpatient basis with the test ferrous sulfate heptahydrate solution containing 20 mg/mL of elemental iron. The treatment was administered orally with the graduated pipette provided. The prescribed initial daily dosage was 2 mg/kg/day,^1^, ^3^ to be administered once a day. The dose administered was calculated using the child’s weight measured at screening and was not modified in response to weight changes during the study. If any gastrointestinal disorders were reported to the investigators during the treatment period (by phone or at a visit), the daily dose was to be divided into two intakes; if the intolerance persisted, it was to be reduced to 1 mg/kg. Other iron formulations, for use by the children or by mothers who were breastfeeding, were the only concomitant treatments prohibited during the study period.

### Study procedures

**Fig. 1:**
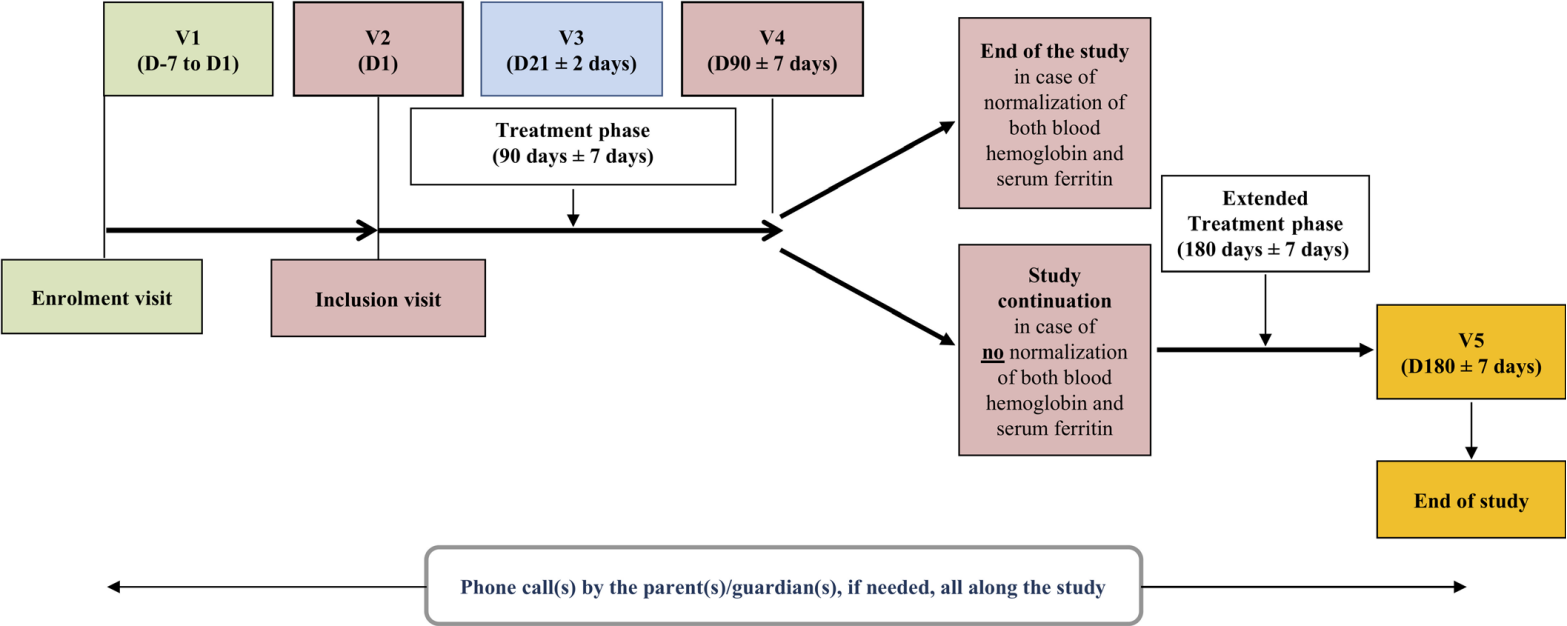
Study scheme. D, day; V, visit.

The study took place over a principal study period of 3 months and included one enrolment visit (V1), which was followed 1 to 7 day(s) later by an inclusion visit (V2), one follow‐up visit 3 weeks (21 ± 2 days) after initiation of the treatment (V3), and another follow‐up visit at the end of the 3–month (90 ± 7 days) treatment period (V4) (Figure 1). At V1, the investigators carried out a physical and clinical examination of the patients, performed the necessary blood tests to confirm the diagnosis of mild‐to‐moderate IDA, collected the medical and surgical history and demographic data, and checked all nonlaboratory eligibility criteria. At V2, the investigators confirmed the eligibility criteria had been met and provided the treatment to the parents / guardians of the included patients. At V3 (week 3), the investigators checked the efficacy and tolerability of the treatment at their own discretion. As a guideline, an increase in blood Hb levels of at least 0.05 g/dL from baseline was considered as a marker of treatment efficiency; this was not enforced as a rule. If deemed appropriate, the treatment was continued up to the end of the 3‐month period. At V4 (month 3), the patients for whom both blood Hb and serum ferritin levels had normalized stopped the treatment. All other patients continued the treatment for another period of 3 months and a supplementary follow‐up visit (V5) was performed 6 months (180 days ± 7 days) after initiation of the treatment. An electronic case report form (e‐CRF) was used to record all patient data. The e‐CRF was fully validated, complied with ICH‐GCP requirements and European regulations, and included a traceability system for data corrections and deletions (audit trail).

### Efficacy outcome measures

The primary outcome measure was the blood Hb level at month 3. Secondary outcome measures included the blood Hb levels at week 3 and month 6; serum ferritin levels at week 3, month 3 and month 6; and Hb response (i.e., normalization of blood Hb levels to ≥11.0 g/dL), ferritin response (i.e., normalization of serum ferritin levels to ≥12 ng/mL), and combined Hb and ferritin response at month 3 and month 6.

### Overall satisfaction with the treatment

Acceptability of the formulation was assessed using a questionnaire filled in by the parents / guardians to evaluate the taste, the ease of administration, and the tolerance of the study treatment. Global satisfaction with the treatment and ease of dose adaptation was assessed by the investigators using two independent questionnaires. The questionnaires were issued at all follow‐up visits and used five‐point rating scales from 1 “very good / very satisfied / very easy” to 5 “not good / not satisfied / not easy at all,” respectively.

### Extent of exposure and treatment compliance

Extent of exposure was calculated as the number of days during which the patient was exposed to the treatment. Treatment compliance was assessed for each patient by measuring the weight of dispensed and returned study treatment bottles. Compliance (%) was expressed as the ratio of the actual weight of the treatment taken (total weight of dispensed bottles minus total weight of returned bottles) to the theoretical weight of the treatment taken × 100.

### Safety assessment

Adverse events (AEs) were recorded throughout the study, and for 30 days after the end of treatment for serious AEs (i.e., events that were life‐threatening, resulting in death or in persistent or significant disability / incapacity, or requiring / prolonging hospitalization). Adverse events were coded using the MedDRA dictionary, version 19.1. A physical examination, including assessment of vital signs (blood pressure and heart rate), and laboratory tests (including blood Hb and serum ferritin levels), was performed at each visit, i.e., V1 to V4 or V5 and at a premature withdrawal visit, when applicable.

### Determination of the sample size

The sample‐size calculation for the study aimed to achieve an accurate confidence interval (CI) for the blood Hb level at month 3. It was based on a two‐sided *α* risk of 5%, a power of 90%, a standard deviation (SD) of 1.1 g/dL,^11^ a CI half‐width of 0.4 g/dL (the noninferiority margin determined by the study of Zaim *et al.* ^12^), and a dropout rate of 20%. According to these hypotheses, 50 patients needed to be included.

### Statistical analysis

SAS® software, version 9.4 (SAS Institute Inc., NC, USA), was used for all statistical analyses. Descriptive statistics were used to analyze all results and no other statistical tests were performed. Qualitative variables were expressed as numbers and percentages of patients; quantitative variables were expressed as the mean ± SD, median, min–max values and 95% CI, as applicable. The full analysis set (FAS), composed of all patients who took at least one dose of the study treatment, was analyzed for safety outcomes. The modified FAS (mFAS), used for efficacy analyses, was a subset of the FAS composed of all patients who met the eligibility criteria. The per protocol set (PPS), used for supportive analysis of the primary outcome measure, was a subset of the FAS composed of all patients without any major protocol deviation or other source of bias for primary outcome measure analysis, and with a minimal treatment exposure of 83 days. In case of premature withdrawal at or after week 3, the observed cases approach was used for all outcome measures, with the premature withdrawal visit replacing the first unobserved scheduled visit (i.e., the month 3 or month 6 visit). Other missing values were not substituted by estimated values for the statistical analysis.

## Results

### Patient disposition

**Fig. 2:**
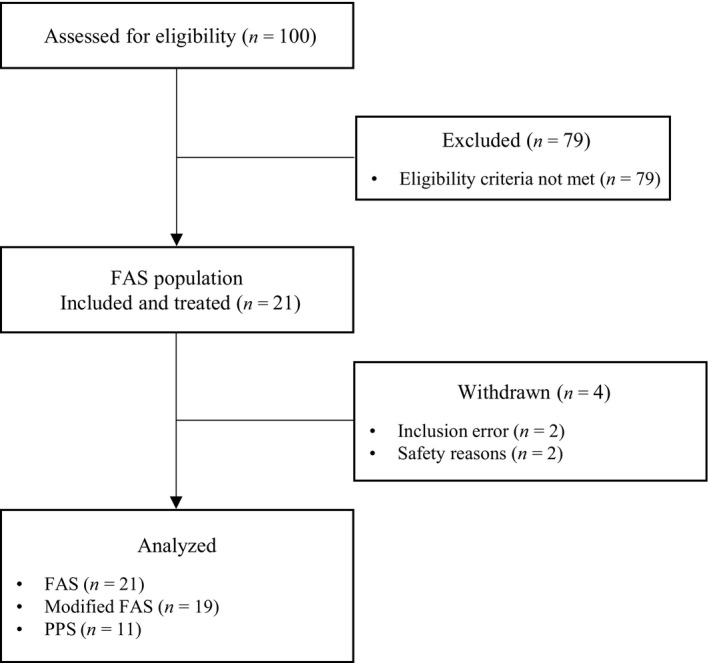
Flow diagram of study participants. Excluded (*n* = 2): inclusion error. Excluded (*n* = 10): patients with major protocol deviation or other source of bias for primary outcome measure analysis. FAS, full analysis set; PPS, per protocol set.

Study enrollment was stopped prematurely on July 31, 2018, due to recruitment difficulties. The patient flowchart is presented in Figure 2. Of the 100 young children screened between June 2016 and July 2018, 21 were included in 6 of the 10 active centers. Thirteen of these participants were included in one single center. All included patients received the study treatment and were included in the FAS. Four patients were prematurely withdrawn from the study, all at or after week 3. Reasons for premature withdrawal were an inclusion error (serum ferritin level >12 ng/mL; *n* = 2) or a safety reason (*n* = 2; including one drug‐related AE). None of the withdrawals were due to a lack of efficacy of the study treatment. All patients received the initial prescribed daily dosage (2 mg/kg/day), in one (*n* = 18) or two (*n* = 3) intakes. The two patients with baseline ferritin level >12 ng/mL who were wrongly included were excluded from the efficacy analysis. Nineteen patients were therefore included in the mFAS.

Overall, 10 patients were excluded from the PPS. As the results obtained for the PPS analysis were similar to those of the FAS and mFAS analyses, only the results for the FAS and mFAS are reported here.

### Patient characteristics at baseline

The demographic and IDA characteristics of the FAS and mFAS patients at baseline are summarized in Table 1. All patients were white infants or toddlers with a mean age of 10.4 months (range: 6–17 months). Most were male (81%), and the majority were not breastfed (57%). At baseline, blood Hb and serum ferritin levels ranged between 8.8 and 10.9 g/dL and 1.0 and 100.5 ng/mL, respectively. Two patients with serum ferritin level >12 ng/mL did not meet the eligibility criteria and were excluded from the efficacy analysis. The characteristics of the mFAS patients were similar to those of the FAS patients.

**Table 1:** Demographic and IDA characteristics of the FAS and modified FAS (mFAS)^†^ at baseline^‡^

Demographic and IDA characteristics	FAS *n* = 21	mFAS^†^ *n* = 19
Age (months), mean ± SD	10.4 ± 3.9	10.7 ± 3.9
Gender, *n* (%)		
Male	17 (81)	16 (84)
Female	4 (19)	3 (16)
Race, *n* (%)		
Asian	0 (0)	0 (0)
Black	0 (0)	0 (0)
White	21 (100)	19 (100)
Other	0 (0)	0 (0)
Weight (kg), mean ± SD	9.5 ± 1.8	9.7 ± 1.8
Height (cm), mean ± SD	75.2 ± 5.4	75.6 ± 5.4
Breastfeeding, *n* (%)		
No	12 (57)	11 (58)
Yes	9 (43)	8 (42)
Severity of IDA, *n* (%)		
Mild	13 (62)	11 (58)
Moderate	8 (38)	8 (42)
Blood hemoglobin level (g/dL), mean ± SD	10.0 ± 0.7	10.0 ± 0.8
Serum ferritin level (ng/mL), median [Q1;Q3]	6.6 [5.0;10.0]	6.5 [4.0;9.0]

FAS, full analysis set; IDA, iron‐deficiency anemia; mFAS, modified full analysis set; Q1; Q3, interquartile range; SD, standard deviation.

^†^ Subset of the FAS composed of all patients who met the eligibility criteria.

^‡^ Inclusion (or screening if missing at inclusion).

### Blood hemoglobin level at month 3 (primary outcome measure)

**Fig. 3:**
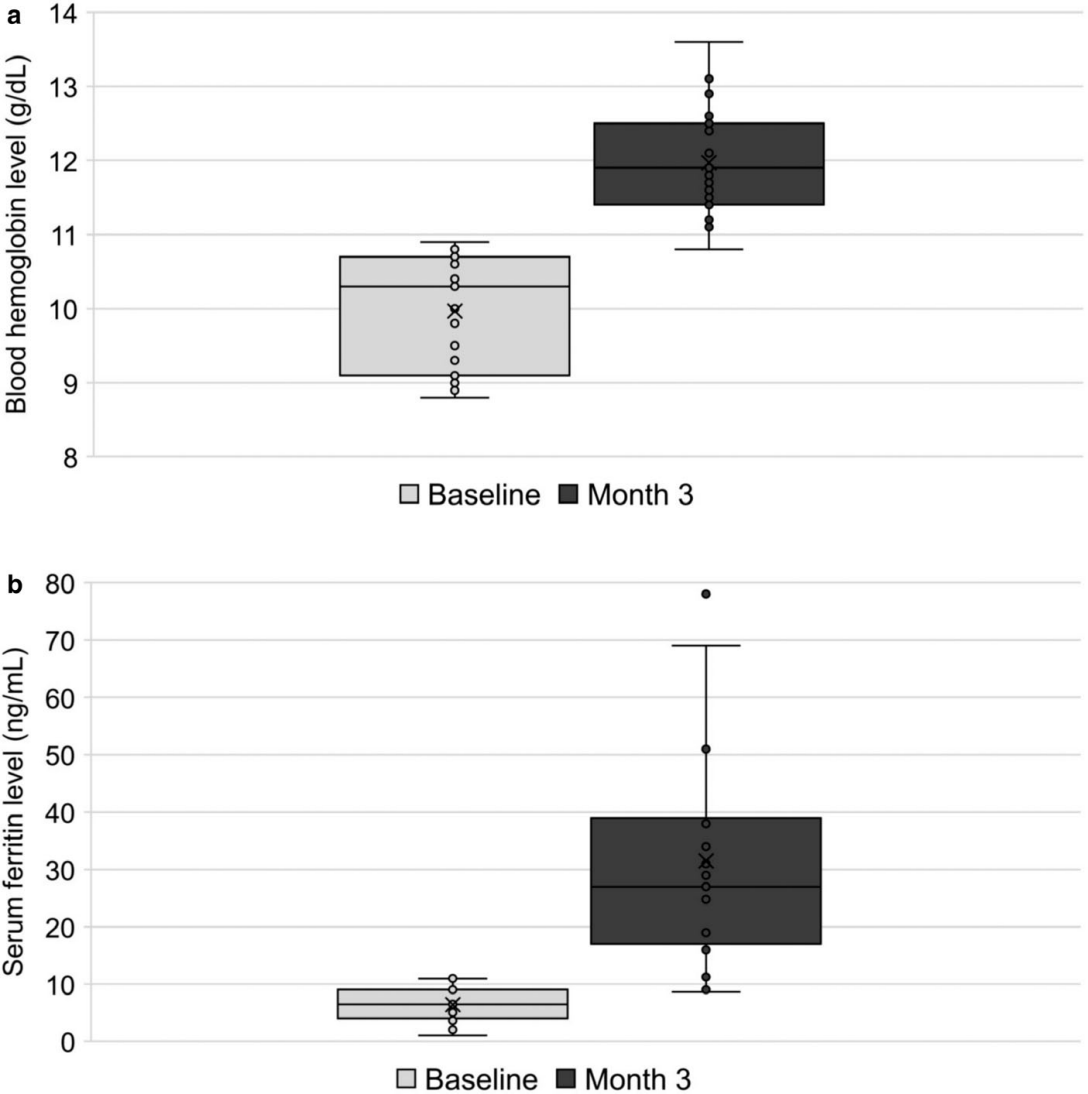
Box plots of blood hemoglobin (a) and serum ferritin (b) levels at baseline and month 3 (*n* = 19).

For the 19 patients of the mFAS, the median exposure was 92 days (range: 88–114 days). The mean ± SD blood Hb level was 12.0 ± 0.7 g/dL with a mean ± SD change of 2.0 ± 1.2 g/dL from baseline. The 95% CI of the mean was [11.6;12.3] g/dL, with a CI half width of 0.4 g/dL. Box plots of the values at baseline and month 3 are shown in Figure 3a.

### Secondary outcome measures

For the 19 patients of the mFAS, the mean ± SD changes in blood Hb and serum ferritin levels between baseline and week 3 were 1.2 ± 0.8 g/dL and 18.4 ± 16.0 ng/mL, respectively. A total of 18 patients (95%) had an increase in blood Hb level of at least 0.05 g/dL from baseline.

At month 3, the mean ± SD serum ferritin level was 31.5 ± 19.4 ng/mL, with a mean ± SD change from baseline of 25.1 ± 20.6 ng/mL; box plots of the values at baseline and month 3 are shown in Figure 3b. Blood Hb and serum ferritin levels had normalized by month 3 in 18 (94.7%) and 16 (84.2%) of the 19 patients of the mFAS, respectively. Among the 17 patients not withdrawn at month 3, only one patient had ferritin levels that had not normalized and therefore continued the treatment up to month 6. The ferritin levels in this patient increased from 6.6 ng/mL at baseline to 11.1 ng/mL at month 3 but did not reach the normalization threshold at month 6 (11.3 ng/mL).

### Extent of exposure and treatment compliance

The mean ± SD extent of exposure of the 21 FAS patients was 89.2 ± 17.6 days, ranging from 22 to 114 days during the 3‐month period. All but two of the 18 patients with compliance data at month 3 had an individual compliance rate of 85% or more.

### Overall satisfaction with the treatment

Parents / guardians rated the taste, ease of administration and tolerance of the study treatment as “good” or “very good” at week 3 (*n* = 14/20; 70.0%) and month 3 (*n* = 17/21; 80.9%). The investigators were “satisfied” or “very satisfied” with the efficacy of the tested treatment in 84.2% (*n* = 16/19) of cases at week 3 and 89.5% (*n* = 17/19) of cases at month 3. None of the investigators reported being “not satisfied” or “not satisfied at all.” Moreover, all the investigators evaluated the adaptation of the dose with the measuring pipette as being “easy” or “very easy” both at week 3 and month 3.

### Safety

Overall, seven of the 21 FAS patients (33.3%) experienced at least one AE during the treatment period (Table 2). Adverse events in the “infections and infestations” system organ class were the most frequently reported (Table 2). With the exception of one case of severe *exanthema subitum*, all AEs were of mild or moderate intensity. Adverse events led to definitive study treatment discontinuation in two patients (9.5%): one (4.8%) as a result of moderate upper abdominal pain, and one (4.8%) due to mild rotavirus gastroenteritis. Both events resolved and the patients recovered. The moderate upper abdominal pain was the only AE suspected of being drug related (Table 2). No serious AEs were reported.

**Table 2:** Adverse events by system organ class and preferred term – full analysis set

	FAS *n* = 21
Patients with at least one drug‐related adverse event, *n* (%)	1 (4.8)
Gastrointestinal disorders, *n* (%)	1 (4.8)
Abdominal pain, upper	1 (4.8)
Patients with at least one non–drug‐related adverse event, *n* (%)	7 (33.3)
General disorders and administration site conditions, *n* (%)	1 (4.8)
Pyrexia	1 (4.8)
Infections and infestations, *n* (%)	7 (33.3)
Bronchitis	1 (4.8)
*Exanthema subitum*	1 (4.8)
Gastroenteritis rotavirus	1 (4.8)
Laryngitis viral	1 (4.8)
Respiratory tract infection	2 (9.5)
Upper respiratory tract infection	2 (9.5)
Urinary tract infection	1 (4.8)
Viral rash	1 (4.8)
Skin and subcutaneous tissue disorders, n (%)	1 (4.8)
Dermatitis allergic	1 (4.8)

## Discussion

Our results show that the new ferrous sulfate heptahydrate solution (20 mg/mL of elemental iron) was efficacious and well tolerated in infants and toddlers at a daily dose of 2 mg/kg for 3 months. At month 3, the mean increase in blood Hb level was 2.0 g/dL, leading to normalization of blood Hb levels in 95% of the treated and evaluable patients and in 100% of the patients completing the 3‐month treatment. None of the premature withdrawals were due to a lack of efficacy of the treatment. Our results are in agreement with those of other studies of daily oral iron supplementation performed on similar populations (i.e., those involving preschool children with mild‐to‐moderate IDA), and were even better than expected. Indeed, in the study by Hawamdeh *et al*., normalization of Hb levels was reached in 78% of the patients.^13^ Moreover, much higher doses of elemental iron (5 mg/kg/day of elemental iron as ferrous sulfate^14^ and 6 mg/kg/day of elemental iron in suspension form)^13^, ^15^ were required in three of these previous studies to obtain a Hb response similar to that observed in our study after 3 months of treatment. The mean Hb levels at baseline were also lower in these previous studies than in the current study, and it has been shown that the lower the Hb level at baseline, the greater the increase after iron supplementation.^16^, ^17^ Some studies reported a smaller increase in the Hb level after administration of a similar^16^, ^18^ or a higher dose^19^ of elemental iron in the form of ferrous sulfate for 2 months: the mean increase in the Hb level observed at month 2 in the studies by Angeles *et al.* and Zlotkin *et al*. was similar to that observed at week 3 in the present study (1.2 g/dL).^18^, ^19^

Our study also showed that the new ferrous sulfate heptahydrate solution led to normalization of the serum ferritin levels in 84% of the patients analyzed for hematologic outcomes at month 3 and in 94% of the patients completing the 3‐month treatment. The mean change in serum ferritin levels from baseline in our study was higher than expected: it was three times higher than that observed in a previous study performed on a similar population and using the same dose of elemental iron but over a shorter treatment duration (2 months instead of 3).^18^ At week 3, the mean change in the serum ferritin level observed in our study was twice as high as that observed at month 2 in the study by Angeles *et al*., indicating a faster replenishment of iron stores in our study. Another study conducted by Faqih *et al*. reported increases in ferritin to levels around twofold higher than those observed in our study, despite similar increases in Hb.^14^ However, the dose of elemental iron in the form of ferrous sulfate administered for the same treatment duration in the Faqih *et al*. study was much higher (5 mg/kg/day) than that used in our study.^14^

Overall, both the patients’ parents / guardians and the investigators were satisfied with the treatment: most reported that the treatment was efficacious, well tolerated, had a good taste, and that adaptation of the dose was easy.

The high level of efficiency observed in our study was probably related to the high level of adherence to treatment. In turn, these high levels of adherence were likely to be the result of the good tolerance of the ferrous sulfate heptahydrate solution by the patients, despite the fact that young children may be especially sensitive to iron supplementation at the gastrointestinal level, and that the daily dose was taken in only one intake by most (*n* = 18/21) patients treated. One‐third of the patients reported at least one AE, with all but one of these AEs being of mild or moderate intensity. As expected in this study population, infections and infestations were the most frequent AEs reported.^13^, ^18^ Upper abdominal pain, a common adverse reaction to iron supplementation, was the only suspected drug‐related AE and was reported in one patient. However, contrary to other studies, no other gastrointestinal AEs (vomiting, nausea, or diarrhea) were reported in our study.^13^, ^15^, ^18^, ^19^, ^20^ As expected, no serious AEs were reported.

Our study had some limitations. First, the number of patients included was lower than expected (21 included versus the 50 patients planned) due to recruitment difficulties. Although several measures were taken to help with recruitment, these difficulties persisted and were attributed to the moderate rate of anemia (26%) in young children aged 6 to 59 months in Poland^2^ and to the fact that the study involved taking several blood samples in a population of infants and toddlers. The decision was taken to stop study recruitment prematurely on July 31, 2018, after 100 children had been screened. Nevertheless, the attrition rate before inclusion was consistent with expectations (~80%). Moreover, the CI half‐width of the blood Hb level was in accordance with the initial hypotheses, thus confirming the accuracy of the primary outcome CI and compensating for the small sample size of the study. Second, another study limitation was the high percentage of patients excluded from the PPS (48%) due to major protocol deviations or other risks of primary analysis bias. However, these exclusions had no impact on the size of the change in blood Hb levels induced by the treatment. Indeed, the results of the PPS analysis were similar to those of the FAS and mFAS analyses. Finally, the inclusion of 13 patients from the same center may have been a potential source of bias. Nevertheless, the efficacy criteria assessment, based on blood tests, was particularly objective.

Although it had no impact on the study results, it was noticeable that a large majority of the children included in our study were male, with a male‐to‐female ratio of 4:1. Male‐to‐female ratios varying from 1:1 to 2:1 have been reported in previous studies of similar populations.^13^, ^14^, ^15^, ^18^ A large retrospective review conducted by Joo *et al*., evaluating the demographics of 1330 IDA patients aged 6–23 months, confirmed that there is a general trend towards higher male participation in this population with a sex ratio of 2.14:1.^5^ We concluded that the 4:1 sex ratio observed in the present study was partly due to chance.

In conclusion, despite recruitment ending prematurely with about half the expected number of included patients, our study showed that the new ferrous sulfate heptahydrate solution led to complete recovery of anemia and to quasi‐complete iron store replenishment in the patients who completed the 3‐ month treatment. Moreover, both the parents / guardians and the investigators were globally satisfied with the treatment. Finally, our new ferrous sulfate heptahydrate solution exhibited a good tolerability profile, leading to high levels of compliance with treatment. The only drug‐related AE reported was one that had already been identified as potential AE for the study drug. No new safety signal was raised in the evaluated population. Thus, our study demonstrated that a 3‐month treatment with 2 mg/kg/day of Tardyferon® oral solution (20 mg/mL) provides a substantial therapeutic benefit for infants and toddlers suffering from mild or moderate IDA.

## Disclosure

Trial registration: 2015‐000995‐88 (EudraCT number). L. Pachuta Węgier, M. Kubiak, and A. Liebert received a grant from Pierre Fabre Médicament as investigators of this study. T. Clavel received a grant from Pierre Fabre Médicament for consulting activities. A. Montagne, A. Stennevin, S. Roye, and A. Boudribila are employees of Pierre Fabre Médicament. The authors declare no other conflict of interest.

## Author contributions

T.C. participated in the study design conception. L.P.W. was the study coordinating investigator. M.K. and A.L. participated as active investigators. A.S. and A.B. respectively monitored the operational and medical activities of the study. S.R. supervised the analysis of data. A.M. supervised the study report and manuscript drafting. All authors read and approved the final manuscript.
